# Supplementary material for: Resolving the full spectrum of human genome variation using Linked-Reads
Source: Genome Res. 2019 Apr;29(4):635–45. doi: 10.1101/gr.234443.118 (PMC6442396; doi:10.1101/gr.234443.118)
Supplement: Supplemental Material [file supp_gr.234443.118_Supplemental_longranger-2.2.zip › longranger-2.2.tar.bz2!/lr2.2-public/lib/stan/cmdstan/stan/doc/api/html/functions__grammar_8hpp_source.html]

Stan: src/stan/gm/grammars/functions\_grammar.hpp Source File


|  |  |
| --- | --- |
|  | Stan  2.5.0  probability, sampling & optimization |


- Main Page
- Related Pages
- Namespaces
- Classes
- Files

- File List
- File Members

All Classes Namespaces Files Functions Variables Typedefs Enumerations Enumerator Friends Macros Pages

- src
- stan
- gm
- grammars

functions\_grammar.hpp

Go to the documentation of this file.

1 #ifndef STAN\_\_GM\_\_PARSER\_\_FUNCTIONS\_GRAMMAR\_HPP\_\_

2 #define STAN\_\_GM\_\_PARSER\_\_FUNCTIONS\_GRAMMAR\_HPP\_\_

3

4 #include <set>

5 #include <string>

6 #include <sstream>

7 #include <vector>

8

9 #include <boost/spirit/include/qi.hpp>

10

11 #include <stan/gm/ast.hpp>

12 #include <stan/gm/grammars/whitespace\_grammar.hpp>

13 #include <stan/gm/grammars/statement\_grammar.hpp>

14 #include <stan/gm/grammars/bare\_type\_grammar.hpp>

15

16 namespace stan {

17

18  namespace gm {

19

20  template <typename Iterator>

21  struct functions\_grammar

22  : boost::spirit::qi::grammar<Iterator,

23  std::vector<function\_decl\_def>(),

24  whitespace\_grammar<Iterator> > {

25

26  // global variable info

27  variable\_map& var\_map\_;

28

29  // local info to keep track of which functions declared defined

30  // so far

31  std::set<std::pair<std::string,

32  function\_signature\_t> > functions\_declared\_;

33  std::set<std::pair<std::string,

34  function\_signature\_t> > functions\_defined\_;

35

36  std::stringstream& error\_msgs\_;

37

38  // grammars

39  statement\_grammar<Iterator> statement\_g;

40  bare\_type\_grammar<Iterator> bare\_type\_g;

41

42  // constructor

43  functions\_grammar(variable\_map& var\_map,

44  std::stringstream& error\_msgs);

45

46  // rules

47  boost::spirit::qi::rule<Iterator,

48  std::vector<function\_decl\_def>(),

49  whitespace\_grammar<Iterator> >

50  functions\_r;

51

52  boost::spirit::qi::rule<Iterator,

53  boost::spirit::qi::locals<bool,int>,

54  function\_decl\_def(),

55  whitespace\_grammar<Iterator> >

56  function\_r;

57

58  boost::spirit::qi::rule<Iterator,

59  std::vector<arg\_decl>(),

60  whitespace\_grammar<Iterator> >

61  arg\_decls\_r;

62

63  boost::spirit::qi::rule<Iterator,

64  arg\_decl(),

65  whitespace\_grammar<Iterator> >

66  arg\_decl\_r;

67

68  boost::spirit::qi::rule<Iterator,

69  std::string(),

70  whitespace\_grammar<Iterator> >

71  identifier\_r;

72

73  };

74

75

76  }

77 }

78

79

80 #endif

stan::gm::functions\_grammar::bare\_type\_g

bare\_type\_grammar< Iterator > bare\_type\_g

**Definition:** functions\_grammar.hpp:40

stan::gm::functions\_grammar::arg\_decls\_r

boost::spirit::qi::rule< Iterator, std::vector< arg\_decl >), whitespace\_grammar< Iterator > > arg\_decls\_r

**Definition:** functions\_grammar.hpp:61

bare\_type\_grammar.hpp

stan::gm::statement\_grammar

**Definition:** statement\_2\_grammar.hpp:19

stan::gm::function\_decl\_def

**Definition:** ast.hpp:776

statement\_grammar.hpp

stan::gm::functions\_grammar::arg\_decl\_r

boost::spirit::qi::rule< Iterator, arg\_decl(), whitespace\_grammar< Iterator > > arg\_decl\_r

**Definition:** functions\_grammar.hpp:66

stan::gm::variable\_map

**Definition:** ast.hpp:439

stan::gm::functions\_grammar::var\_map\_

variable\_map & var\_map\_

**Definition:** functions\_grammar.hpp:27

stan::gm::functions\_grammar::function\_r

boost::spirit::qi::rule< Iterator, boost::spirit::qi::locals< bool, int >, function\_decl\_def(), whitespace\_grammar< Iterator > > function\_r

**Definition:** functions\_grammar.hpp:56

stan::gm::functions\_grammar::functions\_grammar

functions\_grammar(variable\_map &var\_map, std::stringstream &error\_msgs)

**Definition:** functions\_grammar\_def.hpp:290

stan::gm::functions\_grammar::statement\_g

statement\_grammar< Iterator > statement\_g

**Definition:** functions\_grammar.hpp:39

stan::gm::functions\_grammar::functions\_declared\_

std::set< std::pair< std::string, function\_signature\_t > > functions\_declared\_

**Definition:** functions\_grammar.hpp:32

stan::gm::functions\_grammar::functions\_r

boost::spirit::qi::rule< Iterator, std::vector< function\_decl\_def >), whitespace\_grammar< Iterator > > functions\_r

**Definition:** functions\_grammar.hpp:50

stan::gm::functions\_grammar::identifier\_r

boost::spirit::qi::rule< Iterator, std::string(), whitespace\_grammar< Iterator > > identifier\_r

**Definition:** functions\_grammar.hpp:71

ast.hpp

stan::gm::functions\_grammar::functions\_defined\_

std::set< std::pair< std::string, function\_signature\_t > > functions\_defined\_

**Definition:** functions\_grammar.hpp:34

stan::gm::whitespace\_grammar

**Definition:** whitespace\_grammar.hpp:11

whitespace\_grammar.hpp

stan::gm::bare\_type\_grammar

**Definition:** bare\_type\_grammar.hpp:19

stan::gm::functions\_grammar::error\_msgs\_

std::stringstream & error\_msgs\_

**Definition:** functions\_grammar.hpp:36

stan::gm::functions\_grammar

**Definition:** functions\_grammar.hpp:21

stan::gm::function\_signature\_t

std::pair< expr\_type, std::vector< expr\_type > > function\_signature\_t

**Definition:** ast.hpp:104

stan::gm::arg\_decl

**Definition:** ast.hpp:767


---

[ Stan Home Page ]

*© 2011–2014,
Stan Development Team.*
